# 2A and the Auxin-Based Degron System Facilitate Control of Protein Levels in *Plasmodium falciparum*

**DOI:** 10.1371/journal.pone.0078661

**Published:** 2013-11-13

**Authors:** Andrea Kreidenweiss, Annika V. Hopkins, Benjamin Mordmüller

**Affiliations:** Institute of Tropical Medicine, University of Tübingen, Tübingen, Germany; RWTH Aachen University, Germany

## Abstract

Analysis of gene function in *Plasmodium falciparum,* the most important human malaria parasite, is restricted by the lack of robust and simple reverse genetic tools. Approaches to manipulate protein levels post-translationally are powerful tools to study *protein-off* effects especially in the haploid malaria parasite where genetic knockouts of essential genes are lethal. We investigated if the auxin-inducible degron system is functional in *P. falciparum* and found that degron-tagged yellow fluorescent protein levels were efficiently reduced upon addition of auxin which otherwise had no effect on parasite viability. The genetic components required in this conditional approach were co-expressed in *P. falciparum* by applying the small peptide 2A. 2A is a self-processing peptide from Foot-And-Mouth Disease virus that allows the whole conditional system to be accommodated on a single plasmid vector and ensures stoichiometric expression levels.

## Introduction

Research on *Plasmodium falciparum*, the most important human parasitic pathogen, is hampered by the lack of simple approaches to study the function of parasite proteins, particularly potential drug and vaccine targets. Approaches which conditionally reduce protein levels may boost knowledge about potential targets, since they allow investigation of proteins that cannot be knocked out.

After many frustrating attempts, genetic manipulation of *P. falciparum* became routine in the last decade. Nevertheless, it is still a cumbersome and challenging procedure, especially when it comes to introduction of more than one gene of interest, which is a prerequisite for all artificially inducible gene activation or repression systems [1]. The parasite’s AT-rich genes and regulatory sequences are unstable in *Escherichia coli*, therefore cloning of vectors is difficult [2]. As a remedy, dual or triple vector transfection strategies are usually used. This approach further lowers the already poor transfection efficiency in *P. falciparum* resulting in stochastic co-expression in single cells as well as unpredictable stoichiometry of protein concentration [3]. Gene co-expression from one plasmid has been facilitated in various cell lines by the use of polycistronic expression vectors containing internal ribosome entry site (IRES) elements. Nevertheless, there are various constraints to their use in *P. falciparum* such as their restriction to specific organisms, their large size (several hundred nucleotides), and unequal expression levels of genes upstream and downstream of the IRES. Furthermore, no naturally occurring functional IRES element of *P. falciparum* is known. An alternative, novel approach employs the small peptide 2A (∼20 amino acids) from the Foot-And-Mouth Disease virus (FMDV) in polycistronic expression vectors [4]. This element - when cloned in between two genes in a single open reading frame - results in individual proteins of almost equimolar quantities by a co-translational ribosome ‘skipping’ mechanism [5]. 2A has been applied in many eukaryotic cell types (www.st-andrews.ac.uk/ryanlab/Index.htm) and only recently in *P. falciparum* [6]. Its small size, the cleavage activity independent of external factors, and almost equimolar levels of co-expressed proteins, make 2A a compelling technology for use to study multiple proteins or protein complexes in *P. falciparum*. In addition, 2A technology may improve conditional gene expression systems that generally depend on co-expression of multiple proteins in individual cells.

Conditional protein degradation has only been rarely applied in *P. falciparum* and has not been extensively utilized to address biological questions. To stabilize the target protein and to maintain ‘protein-on’ phenotypes, techniques based on a destabilization domain require continuous drug pressure by a small molecule whose effects on parasites in long-term culture are unknown [7], [8]. An alternative, powerful approach to conditionally influence protein levels is the auxin-inducible degron (AID) system originating from plants [9]. The plant hormone auxin mediates interaction of auxin specific E3 ubiquitin ligase SCF^Tir^ and auxin responsive plant transcription repressors (AUX/IAAs) leading to degradation of the latter by the proteasome. This biotechnological application deploys (1) auxin as a small molecule switch, (2) an auxin-responsive AUX/IAA sequence fused to the protein of interest (AID) and (3) TIR1 as an auxin binding domain of the E3 ubiquitin ligase complex SCF^Tir^ (Skp1–Cullin–F-box protein). TIR1 is plant specific and binds to endogenous Skp1-Cullin complex via its F-box domain [10]. Skp1 is evolutionarily highly conserved amongst eukaryotes whereas hundreds of members are known as the F-box protein super family with all of them sharing a conserved F-box domain.

Here, we show that the AID system can be used in *P. falciparum* to conditionally alter intracellular protein concentrations. To facilitate and control co-expression of AID degron components from one single *P. falciparum* transfection vector, we applied an optimized FMDV 2A element.

## Materials and Methods

### *P. falciparum* Continuous Culture and Transfection

*P. falciparum* laboratory strains 3D7 and D10 were obtained from the Malaria Research and Reference Reagent Resource Center (MR4). Parasites were grown according to Trager and Jensen with minor modifications [11]. Parasite complete culture medium (CCM) was based on RPMI 1640 medium (Sigma Aldrich) supplemented with 2 mM L-glutamine, 24 mM HEPES, 100 µM gentamycin (Gibco), 130 µM hypoxantine and 2.5 g Albumax II. If not otherwise stated chemicals were purchased from Invitrogen. Parasites were gown in O^+^ erythrocytes with the hematocrit adjusted to 3%. CCM was daily changed against pre-warmed, fresh CCM and parasitemia was routinely checked microscopically on a Giemsa-stained (Merck) thin blood smear. Parasitemia was thoroughly kept below 5%. Cultures were grown at 37°C in an incubator (Heracell, Thermo Scientific) with a copper chamber at controlled atmosphere of 5% O_2_, 5% CO_2_ and 90% N_2_. Parasites were routinely screened for mycoplasma contamination by PCR using genus-specific primers covering the five mycoplasma species which account for 95% of all contaminations [12].

If necessary, synchronization of parasites was done when mainly ring stages were present. Parasite culture was pelleted by centrifugation and treated with 5% sterile filtered sorbitol for 5 min at room temperature. After two times washing with CCM, parasite culture was restarted.

To generate transgenic parasites, schizont stage parasites were cultured in fresh human O^+^ erythrocytes preloaded with DNA by electroporation [13]. CCM was enriched with 10% of human serum (Blood Donation Center, Mannheim, Germany) and drug pressure (5 µg/ml blasticidin S or 5 nM WR99210) was started three days later to select for transgenic parasites. All parasites were episomally transfected with the respective plasmid.

### Plasmid Construction

To investigate 2A element functionality in *P. falciparum*, pHGB was chosen as backbone vector to obtain the final transfection vector pHGB_R2Y2B [14]. pHGB contains a plasmodial expression cassette with a small multiple cloning site (MCS) but lacks plasmodial selection markers. By digesting pHGB with BglII and NotI, the single open reading frame *dsred*-*2A*-*eyfp*-*Pf2A*-*bsd* was cloned into the MCS with *dsred* coding for red fluorescent protein (DsRed), *eyfp* coding for enhanced yellow fluorescent protein (eYFP) and *bsd* for blasticidin S-deaminase S (BSD). The sequence of the self-processing element 2A is a N-terminally extended form of the wild-type FMDV 2A sequence (pSTA1/34) with increased cleavage activity (96%) and equimolar ratio of cleavage products [5]. *Pf2A* codes for the same amino acid sequence but with *P. falciparum* codon usage to reduce intra-plasmodial recombination. pDsRed-Monomer-N1 (Clontech) served as template for *dsred* and *eyfp*, an enhanced yellow-green variant of *gfp*, was obtained based on pEYFP-1 vector (Clontech).

Control plasmid pC3_eYFP was constructed by cloning *eyfp* into the multi cloning site of the expression vector pcDNA3 (Invitrogen) for eYFP expression in human HeLa cells.

pCHD 1/2 was chosen as the plasmid backbone for *P. falciparum* expression vector pCHD_T2AY [14]. Using BglII and NotI sites, the insert T2AY was directly cloned into a previously generated pCHD plasmid that contained the expression cassette (with PfHsp86 5′ promoter and PbDT 3′ UTR) from pHGB vector after Gateway cloning and the selection marker human *dhfr*. Parasites successfully transfected by pCHD_T2AY were selected in CCM containing 5 nM WR99210. The insert T2AY was *de novo* synthesized (Eurofins MWG Operon) based on the polycistronic DNA sequence consisting of (T) *Oryza sativa* TIR1 gene c-terminally tagged with 9x c-myc, (2) an optimized 2A sequence of 26 amino acid length (pSTA1/34), (A) the *Arabidopsis thaliana* IAA17 gene as the aid-degron, and (Y) the reporter gene eYFP.

### Fluorescence Microscopy

Non-fixed transgenic parasites were monitored with a Leica DMLB fluorescent microscope using the filters ET-Cy3 F46-004 for detection of DsRed fluorescence and ET-YFP F46-003 for eYFP detection. Photographs were captured with a ProgRes C10^Plus^ camera and the ProgRes Capture Pro 2.1. software.

### Protein Extraction and Western Blotting

*P. falciparum* protein extraction procedure started with initial saponin lysis of red blood cells (RBC) to remove erythrocyte proteins, particularly hemoglobin. Therefore, infected RBCs (iRBC) were treated with PBS containing 0.075% freshly prepared saponin (Sigma) for 5 min at room temperature followed by thorough washing (5–10 times at 4°C) until the supernatant turned clear. Then the parasite pellet was lysed in high salt buffer (20 mM HEPES,350 mM NaCl, 1 mM MgCl, 0,5 mM EDTA, 0,1 mM EGTA, 20% glycerol, 1% NP40, 1M DTT and 1 tablet of complete protease inhibitor cocktail (Roche)) and incubated for 30 min at −20°C. After centrifugation (13000 rpm, 30 min, 4°C), supernatant was transferred into a new tube and 4x SDS loading buffer (125 mM Tris pH6.8, 6% SDS, 20% glycerol, 0,2% bromophenol blue, 10% ß-mercaptoethanol) was added and incubated for 5 min at 95°C. Protein extraction of transfected HeLa cells was done identically but without the initial saponin lysis.

Western blotting was done following standard protocols. eYFP was detected with a GFP-specific monoclonal antibody (Roche) at a final dilution of 1∶1000. The blot was stripped and re-probed with an anti-α UE proteasome antibody (Biomol, clone MCP231) at 1∶500 to control for equal protein loading. Neither DsRed-specific antibody (L-18) from Santa Cruz nor DsRed-specific antibody from Clontech was able to detect DsRed expressed by *P. falciparum*. Both antibodies were tested at 1∶1000 and 1∶500 final dilutions. TIR1-myc was identified by c-Myc-specific antibody (Santa Cruz) applied at 1∶1000 final dilution.

Protein lysates of D10_ACP-GFP parasites (kindly provided by Geoffrey I. McFadden) and HeLa cells expressing eYFP served as controls for eYFP expression.

### Susceptibility of Parasites to Auxin and its Derivatives

The natural phytohormone indole-acetic acid (IAA, auxin) and the synthetic auxins 1-naphthaleneacetic acid potassium salt (NAA) and 2,4-Dichlorophenoxyacetic acid sodium salt monohydrate (2,4-DAA) were purchased from Sigma Aldrich and dissolved in RPMI 1640 to obtain stock solutions of 100 mM or 10 mM for 2,4-DAA. Chloroquine (CQ) (Sigma Aldrich) served as internal assay control and was diluted from a 10 µM stock solution in water. After sterile filtration, aliquots of all stock solutions were stored at −20°C.

Susceptibility of D10 to the compounds used for inducible degradation was tested as previously published [15]. Briefly, 96 well micro titre culture plates were pre-dosed with 5 mM IAA, 5 mM NAA, 1.1 mM 2,4-DAA and 200 nM chloroquine and serial two-fold dilutions thereof. A drug sensitivity assay was done with 0.05% ring stage parasites and hematocrit adjusted to 1.5%. After 72 h of incubation, culture plates were frozen and thawed twice to lyse the cells. Parasite growth was assessed by measuring *P. falciparum* histidine rich protein-2 (PfHRP 2) with a commercially available enzyme linked immunosorbent assay (Malaria Ag CELISA, Cellabs), according to the manufacturers’ specifications [16]. Analysis of dose-response curves and calculation of inhibitory concentrations was done using the drc package in R (www.r-project.org).

### Auxin Induction Assay to Reduce AID-eYFP Levels

To measure auxin-induced degradation of AID-tagged eYFP, early schizont stage D10_T2AY parasites were cultured in 48 well plates with the same conditions as described above. CCM was enriched with 10% human serum. IAA, NAA or 2,4-DAA were added at a final concentration of 0.5 mM if not otherwise stated. Proteasome-mediated degradation was blocked by pre-incubation with 0.5 µM epoxomicin (Biomol) for one hour before adding the respective concentration of auxin. The assay was stopped by replacing the medium with a staining solution containing 16.2 µM Hoechst 34580 (Molecular Probes) in CCM for 30 min at 37°C for flow cytometric assessment (BD FACS CANTOII with additional violet laser excitation). Values are expressed as proportions (%) of the median fluorescence intensity of D10_T2AY parasites untreated with auxins at the respective time point of data acquisition.

## Results and Discussion

### Cleavage Activity of 2A in *P. Falciparum*

**Figure 1:**
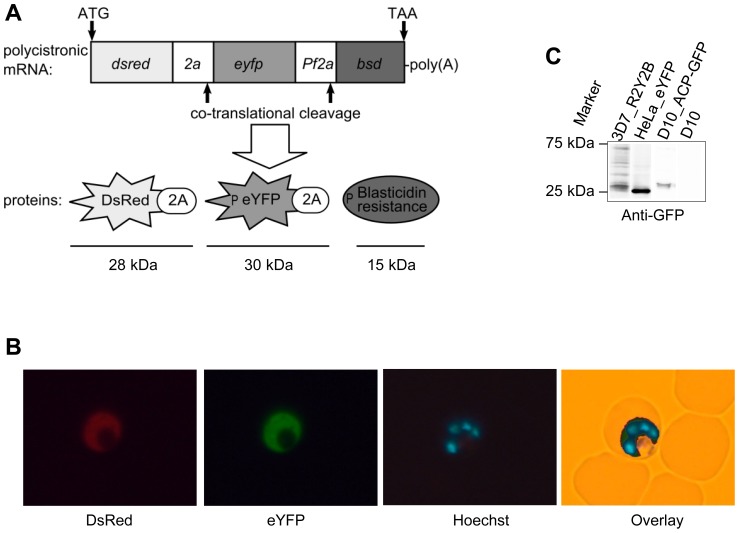
Cleavage activity of 2A in *P. falciparum.* Cleavage activity of 2A in *P. falciparum* was assessed by transgenic parasites (3D7_R2Y2B) expressing A) an open reading frame encoding for DsRed, eYFP and blasticidin-S deaminase (BSD). 2A and *P. falciparum* codon usage adapted version thereof (Pf2A) was placed between the genes. Co-translational cleavage leads to separated proteins C-terminally tagged with 2A and downstream sequences start with a 2A derived proline. B) Fluorescent microscopy showed DsRed and eYFP expressing transgenic parasites (schizont stage) grown under blasticidin selection. Hoechst 33342 staining indicated intraerythrocytic parasites C) Western blot analysis with anti-GFP antibody showed fully cleaved eYFP (30 kDa) and a small proportion of uncleaved polyprotein (73 kDa) of 3D7_R2Y2B parasites. Controls for anti-GFP antibody binding pattern are HeLa cells expressing eYFP (26 kDa), D10_ACP-GFP (32 kDa) and untransfected D10 parasites only. Samples are from two different blots probed with anti-GFP antibody.

The cleavage mechanism of 2A is co-translational and is conserved across many eukaryotes. To test its applicability in co-expression of two and more genes in malaria research, we chose a 24 amino acid long FMDV 2A sequence which has been optimized for high cleavage activity and has been shown to result in equivalent protein levels [5]. To assess its self-processing activity in *P. falciparum,* we created the pHGB based vector pHGB_R2Y2B with a single open reading frame encoding three proteins: red fluorescent protein (DsRed), yellow fluorescent protein (eYFP) and the resistance marker blasticidin S-deaminase (BSD) with 2A elements between the genes (Figure 1A). Codon usage of the 2A elements was different to circumvent recombination during replication.

After transfection with pHGB_R2Y2B, transgenic 3D7_R2Y2B parasites were selected on 5 µg/ml blasticidin S. Only those parasites were able to grow under blasticidin pressure where 2A-mediated co-translational cleavage led to an individual blasticidin S-deaminase protein conferring drug resistance. Fluorescent microscopy showed that transgenic parasites of all stages co-expressed DsRed and eYFP (Figure 1B and Figure S1 - Fluorescence microscopy of 3D7_R2Y2B parasites).

To control for cleavage efficiency of 2A, protein lysates of 3D7_R2Y2B parasites were analyzed by Western blot. Blots were probed with a panel of antibodies against fluorescent proteins. One GFP-specific antibody was sensitive enough to consistently detect a band of 30 kD in 3D7_R2Y2B samples, which corresponds to fully cleaved eYFP (Figure 1C). A weaker band of 73 kD corresponds to the remaining full-length, unprocessed polyprotein. Since cleavage efficiency by 2A is usually not complete in other organisms as well, this finding is not unexpected [4]. D10_ACP-GFP parasites, non-transfected D10 parasites and HeLa cells expressing eYFP served as controls for anti-GFP antibody binding pattern detecting both GFP and its mutant form eYFP.

We conclude that FMDV 2A is fully functional in *P. falciparum* and may be used as a tool for multiple gene co-expression in transgenic parasites, although sequence optimization may lead to even better cleavage efficiency in protozoan parasites.

### Auxin-inducible Degron to Regulate Protein eYFP Levels in *P. Falciparum*

**Figure 2:**
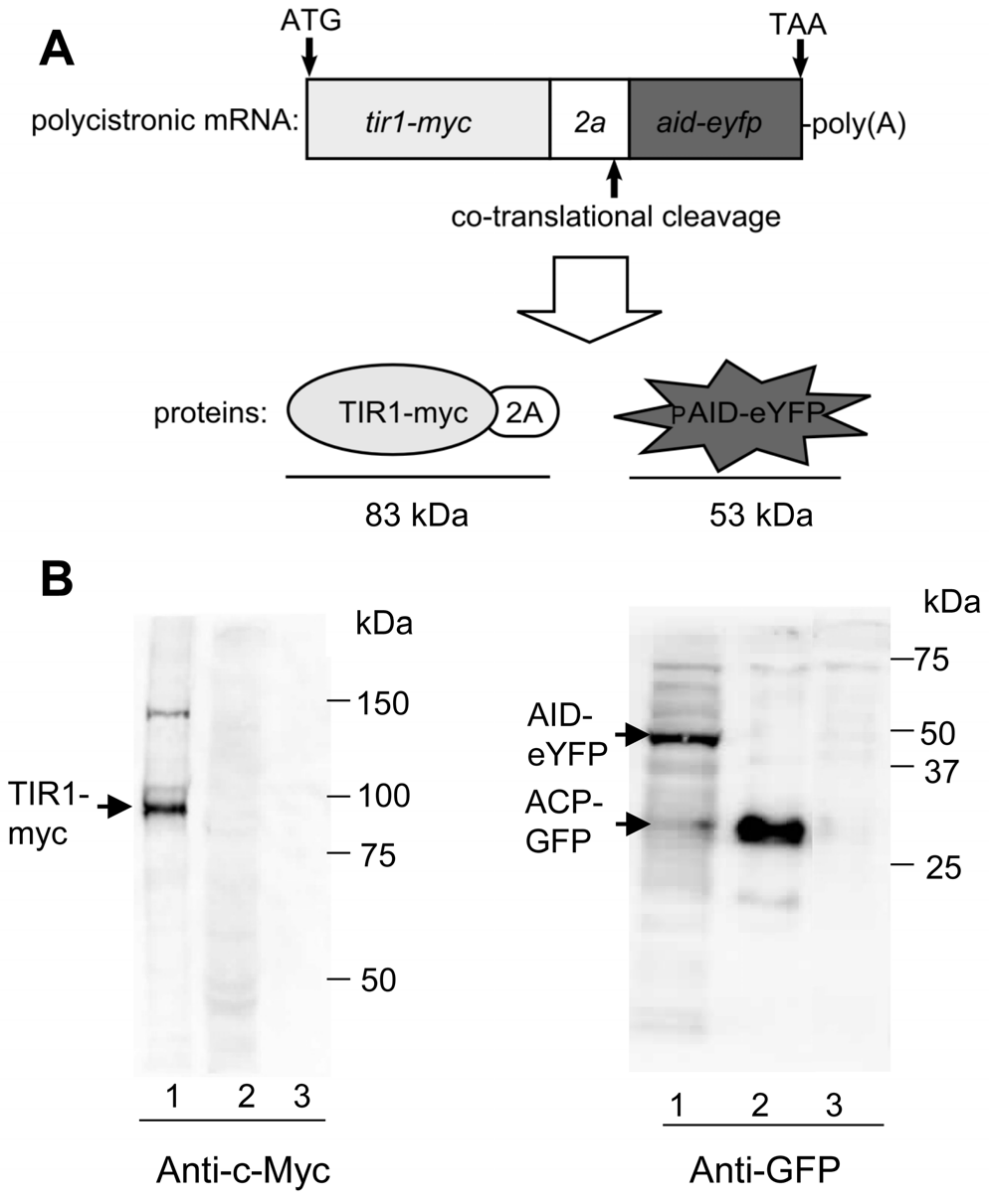
2A mediated co-expression of AID-system functional components in *P. falciparum.* The AID system was realized in *P. falciparum* by transfecting the plasmid pCHD_T2AY resulting in D10_T2AY parasites. A) Scheme of T2AY polycistronic mRNA encoding c-Myc tagged TIR1 and the reporter eYFP N-terminally tagged with AID degron. B) Immunoblots of D10_T2AY protein lysates (without auxin added) to control for expression and correct cleavage of AID system essential components, namely TIR1 and AID-eYFP. Blots with anti-c-Myc antibodies and anti-GFP antibodies were performed separately applying the same parasite protein samples: 1: D10_T2AY, 2: D10_ACP-GFP, D3: D10. D10 and D10_ACP-GFP and are control parasites unresponsive to anti-c-Myc but the latter reactive with anti-GFP antibodies.

As a proof of concept we used 2A in a model system that strictly depends on co-expression of multiple genes in *P. falciparum*. The recently published auxin-inducible degron system was particularly attractive since it would enable conditional protein degradation in *P. falciparum*. Therefore, we transfected D10 parasites with plasmid pCHD_T2AY that contains an open reading frame encoding components necessary for the AID system, namely TIR1 (c-Myc tagged) and eYFP N-terminally tagged with the AID degron and a 2A element between the two proteins (Figure 2A). eYFP was chosen as a reporter for protein depletion because it can be used to measure protein degradation by flow cytometry at the single cell level.

Western blot analysis of D10_T2AY protein lysates with a c-Myc specific antibody showed fully cleaved TIR1 (83 kD) and a small proportion of uncleaved polyprotein (138 kD) (Figure 2B). Anti-GFP antibody detected AID-eYFP fusion protein in the same protein sample of D10_T2AY. D10_ACP-GFP parasites served as positive control for GFP/eYFP detection (ACP-GFP = 32 kD).

The AID system relies on auxin as inducer for protein degradation to result in a ‘protein-off’ phenotype. Initially, we optimized parameters for eYFP protein depletion by investigating natural indole-3-acetic acid (IAA, auxin) and the synthetic auxins 1-naphthaleneacetic acid potassium salt (NAA) and 2,4-Dichlorophenoxyacetic acid sodium salt monohydrate (2,4-DAA) and comparing different concentrations (5 mM, 2.5 mM,1 mM, and 0.5 mM) and incubation times. We found that IAA is superior over NAA and 2,4-DAA, particularly when aiming for a low concentration and a rapid and efficient onset of protein degradation (see Figure S2 - Comparison of auxins for their efficiency in eYFP degradation in *P. falciparum*). Interestingly, eYFP depletion was not increased by prolonged incubation time.

To ensure that auxin itself has no effect on the viability of *P. falciparum*, we measured the inhibitory concentration of IAA, NAA and 2,4-DAA in D10 parasites by culturing the parasites at the respective concentrations for 72 h (see Table 1, and Figure S3– Growth inhibitory curves of auxins in *P. falciparum*). Sensitivity of D10 parasites against chloroquine validated assay performance. IAA proved to be particularly non-toxic for plasmodia and an IAA concentration 10-fold lower than the IC50 was utilised for regulating protein levels (0.5 mM). As the IAA growth inhibitory curve shows, parasites can be grown at this concentration for at least 72 h without any measurable effect on viability. To exclude any potential off-target effect of IAA on the fluorescent protein which could account for an unintended decrease in eYFP levels, D10_ACP-GFP parasites have been treated with IAA and analyzed by flow cytometry (Figure S4 - Off-target effect of IAA on GFP). No effect on GFP levels has been observed and we also exclude off-target effects on eYFP, a mutant of GFP.

**Table 1:** Inhibitory concentrations of auxins against *P. falciparum* strain D10.

	IC50± SD	IC90± SD	IC99± SD
**IAA**	6.4±0.2	14.9±1.4	38.0±6.5
**NAA**	1.4±0.02	3.1±0.3	7.5±1.3
**2,4-DAA**	1.6±0.3	10.0±1.0	76.2±19.1
**CQ**	25±0.6 (nM)	39±9.2 (nM)	65±33.1 (nM)

Each value represents the mean inhibitory concentration (IC) determined from at least three experiments including standard deviation values (SD). Chloroquine (CQ) served as internal control for assay performance. Data are in mM except where otherwise indicated.

Consequently, we assessed if pCHD_T2AY expressing parasites can modulate protein levels upon auxin addition. We grew D10_T2AY parasites in presence of auxin and measured eYFP expression at different time points on single cell level using flow cytometry.

**Figure 3:**
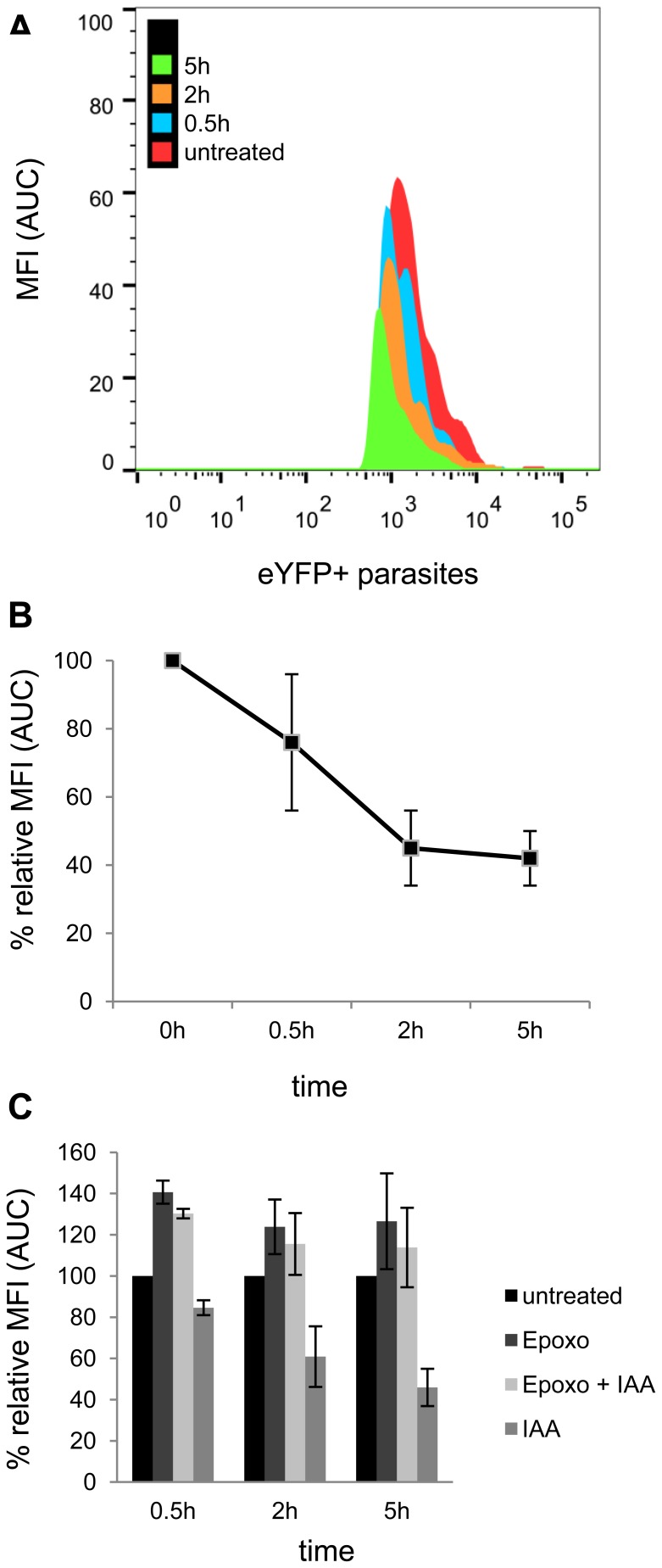
Auxin reduces AID-tagged eYFP levels in *P. falciparum.* D10_T2AY parasites were treated with auxin and eYFP levels were measured by flow cytometry at the respective time points. A) Histogram overlay of eYFP+ parasites of one representative experiment in which D10_T2AY parasites were treated with 0.5 mM IAA for 0.5 h, 2 h and 5 h, respectively, assessed by flow cytometry. B) D10_T2AY treated with 0.5 mM IAA and eYFP median fluorescent intensities (MFI) measured at the respective time point presented as % relative MFI compared to the untreated control. The maximum observed fluorescence intensity of the control was set to 100%. C) D10_T2AY parasites were pretreated with 0.5 µM epoxomicin (1 h) before adding 0.5 mM IAA for 2 h or 5 h, respectively. MFI signals were assessed with flow cytometry. B) and C) represent the MFI and the respective standard deviation bars based on five independently performed experiments. Difference between 0.5 h and 2 h are statistically significant (paired t-test, p<0.005).

To induce protein degradation, D10_T2AY parasites were treated with 0.5 mM IAA for up to 5 h and the number of eYFP positive parasites and their fluorescence intensity was measured by flow cytometry and immunoblotting (Figure S5– Auxin induction assay). A fast and robust down regulation of eYFP was observed upon IAA addition (Figure 3A and 3B). A 1.7-fold reduction of eYFP levels (from 74% to 45%) was achieved between 0.5 h and 2 h after IAA addition (paired t-test p = 0.009). eYFP degradation reached plateau after 2 h (Figure 3B) and remained at 42% of total median fluorescence intensity. The auxin assay is quite robust as seen by the small standard deviation values. To confirm that the ubiquitin proteasome pathway is responsible for degradation of the reporter (AID-tagged eYFP) we pretreated D10_T2AY parasites with proteasome inhibitor epoxomicin for 1 h before adding 0.5 mM IAA. IAA mediated eYFP degradation was abolished completely when epoxomicin was present (Figure 3C). Interestingly, D10_T2AY parasites treated with epoxomicin alone showed a tendency towards higher eYFP levels, which argues for some destabilizing potential of the AID domain per se. Viability of D10_T2AY after epoxomicin treatment was confirmed by parasite life cycle progression after removal of epoxomicin (Figure S6– Viability of D10_T2AY after treatment with epoxomicin).

## Conclusion

In conclusion, we show that the self-processing peptide 2A of FMDV is a valuable genetic tool for co-expression of at least three transgenes in the protozoan parasite *P. falciparum*. In contrast to the ‘2A-like sequence’ of *Thosea asigna* virus applied by Straimer et al. [6], FMDV 2A has been previously shown to be superior in expressing proteins in similar quantities which is of great importance when studying protein interactions where stoichiometry is important. We successfully applied the auxin-inducible degron system in *P. falciparum* and reporter proteins were rapidly and efficiently down-regulated. In contrast to previously reported attempts of conditional protein or gene regulation [1], [7], [8], protein abundance was measured on the single cell level. This is important since it can discriminate between effects on the level of single cells (usually the unit of interest) and the proportion of transgenic cells in the population that may be altered due to the effect of the small molecule used to alter protein concentration. The current system clearly leaves space for optimization, such as more efficient and faster protein degradation as well as application to native plasmodial proteins, but we believe that is a very promising starting point for analysis of proteins that may serve as new targets for antimalarial interventions.

## Supporting Information

Figure S1 **Fluorescence microscopy of 3D7_R2Y2B parasites.**
(TIF)

Figure S2 **Comparison of auxins for their efficiency in eYFP degradation in** ***P. falciparum.***
(TIF)

Figure S3 **Growth inhibitory curves of auxins in** ***P. falciparum.***
(TIF)

Figure S4 **Off-target effect of IAA on GFP.**
(TIF)

Figure S5 **Auxin induction assay.**
(TIF)

Figure S6 **Viability of D10_T2AY after treatment with epoxomicin.**
(TIF)
