# Supplementary figures and images for: 2A and the Auxin-Based Degron System Facilitate Control of Protein Levels in Plasmodium falciparum
Source: PLoS One. 2013 Nov 13;8(11):e78661. doi: 10.1371/journal.pone.0078661 (PMC3827281; doi:10.1371/journal.pone.0078661)

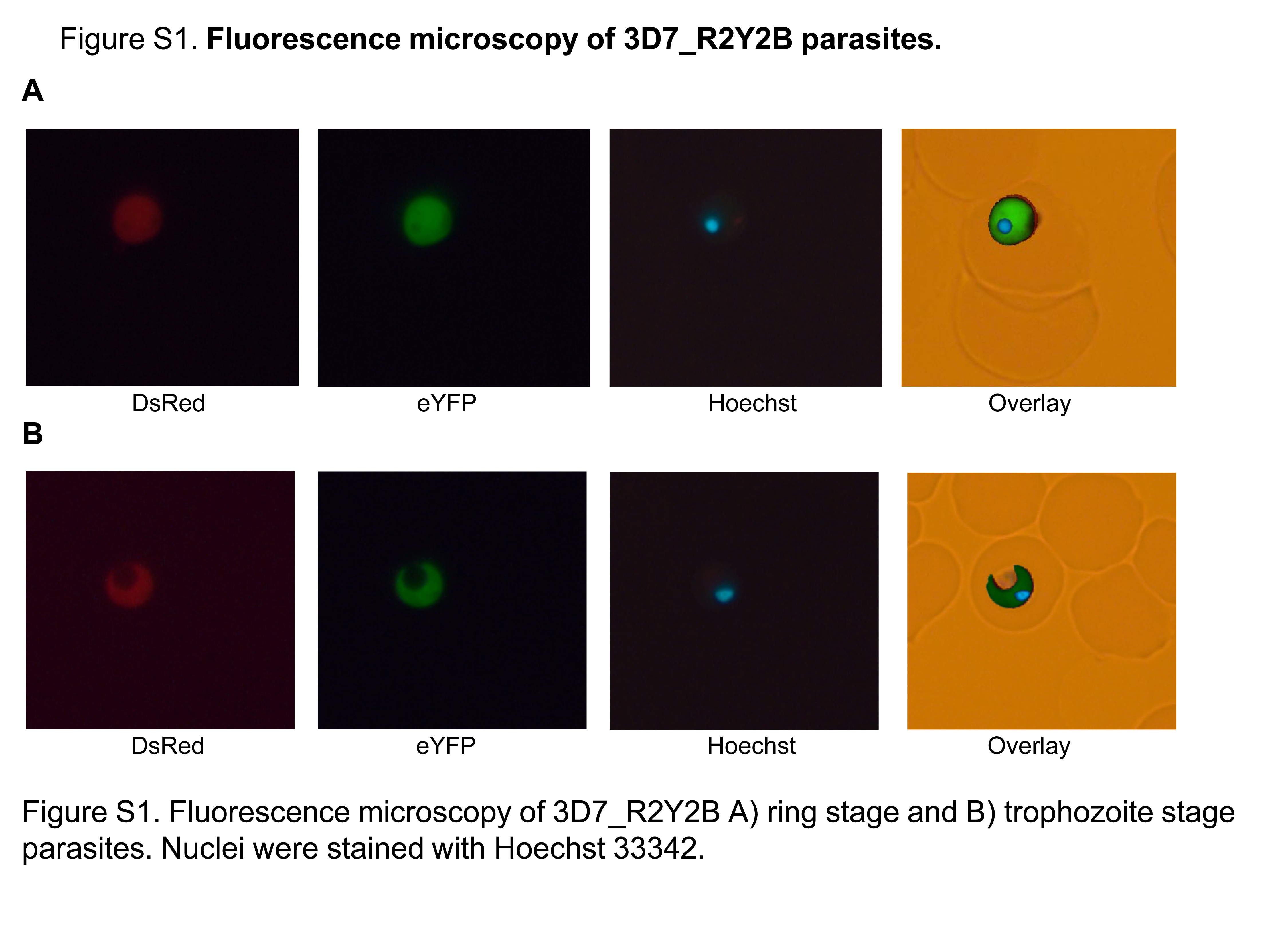

Supplement: Figure S1 — Fluorescence microscopy of 3D7_R2Y2B parasites. (TIF) [file pone.0078661.s001.tif]

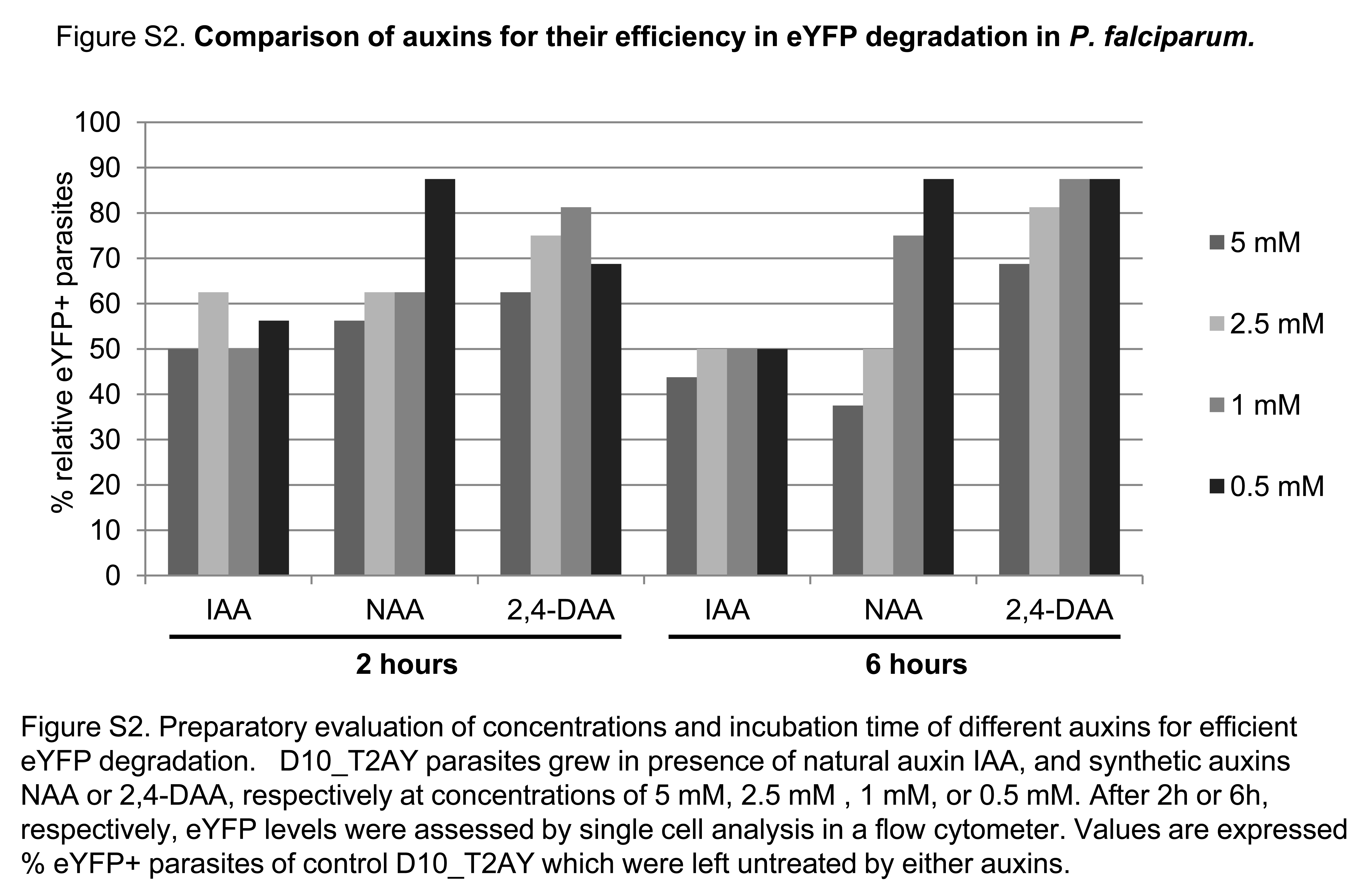

Supplement: Figure S2 — Comparison of auxins for their efficiency in eYFP degradation in P. falciparum. (TIF) [file pone.0078661.s002.tif]

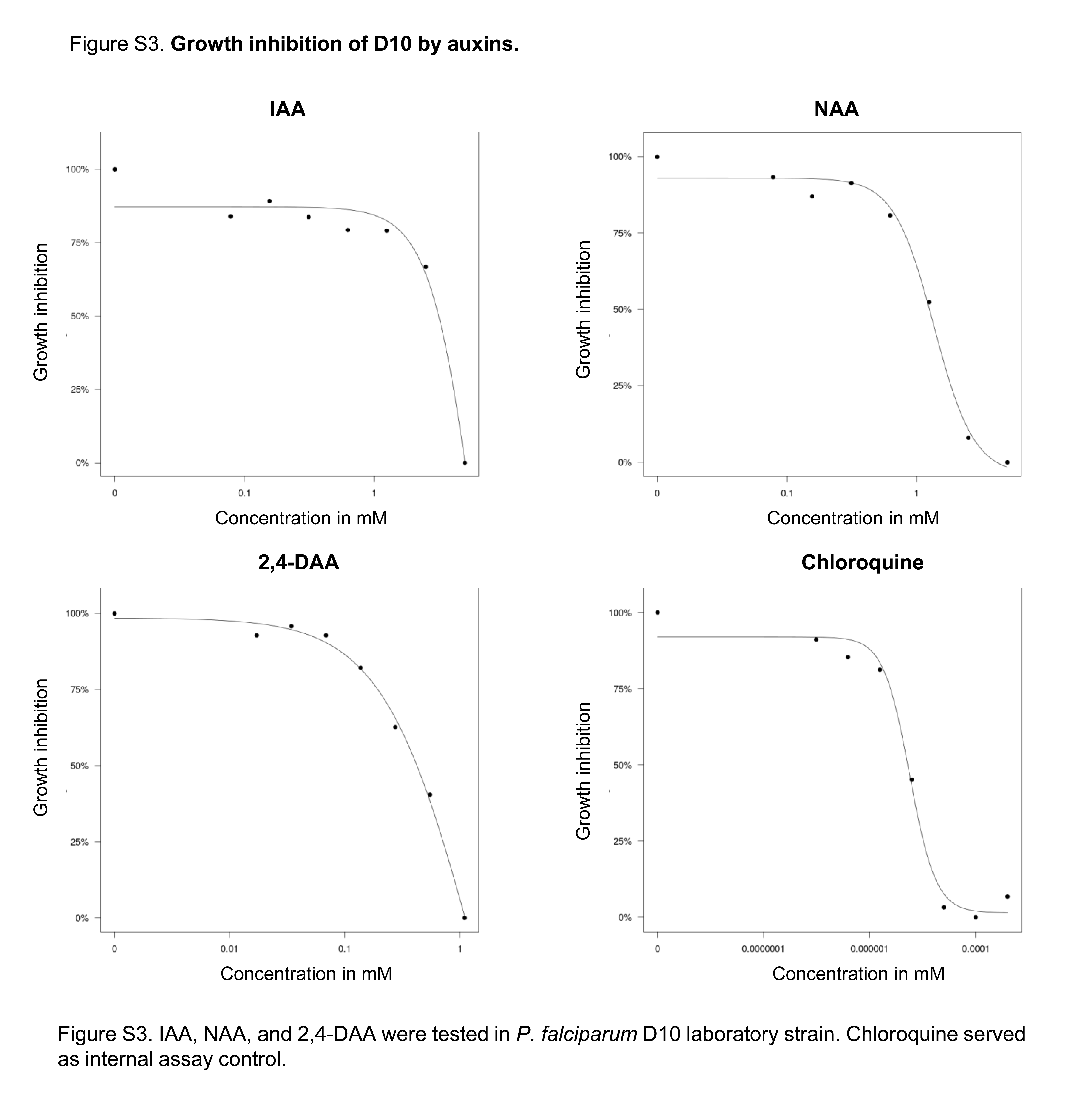

Supplement: Figure S3 — Growth inhibitory curves of auxins in P. falciparum. (TIF) [file pone.0078661.s003.tif]

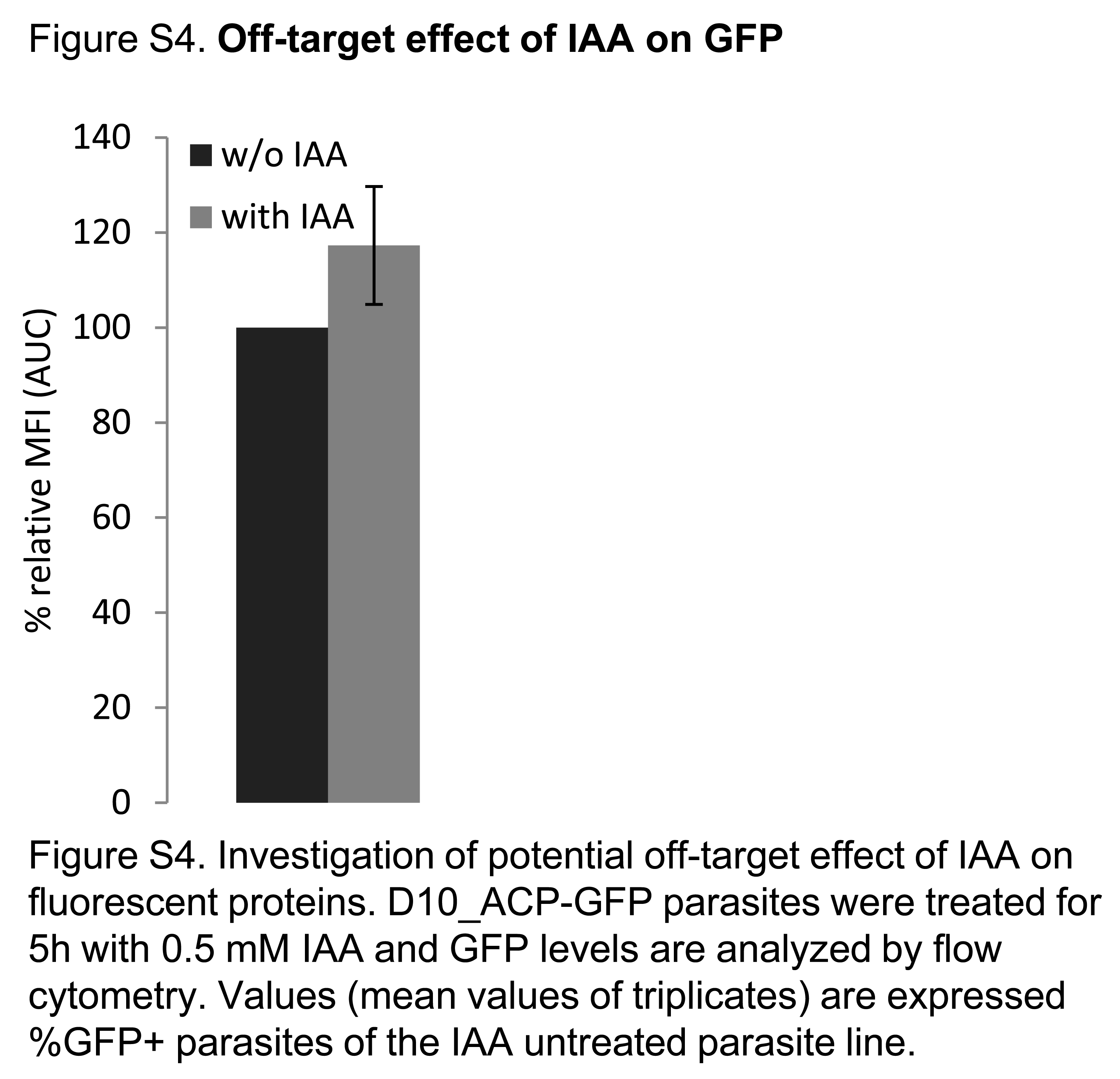

Supplement: Figure S4 — Off-target effect of IAA on GFP. (TIF) [file pone.0078661.s004.tif]

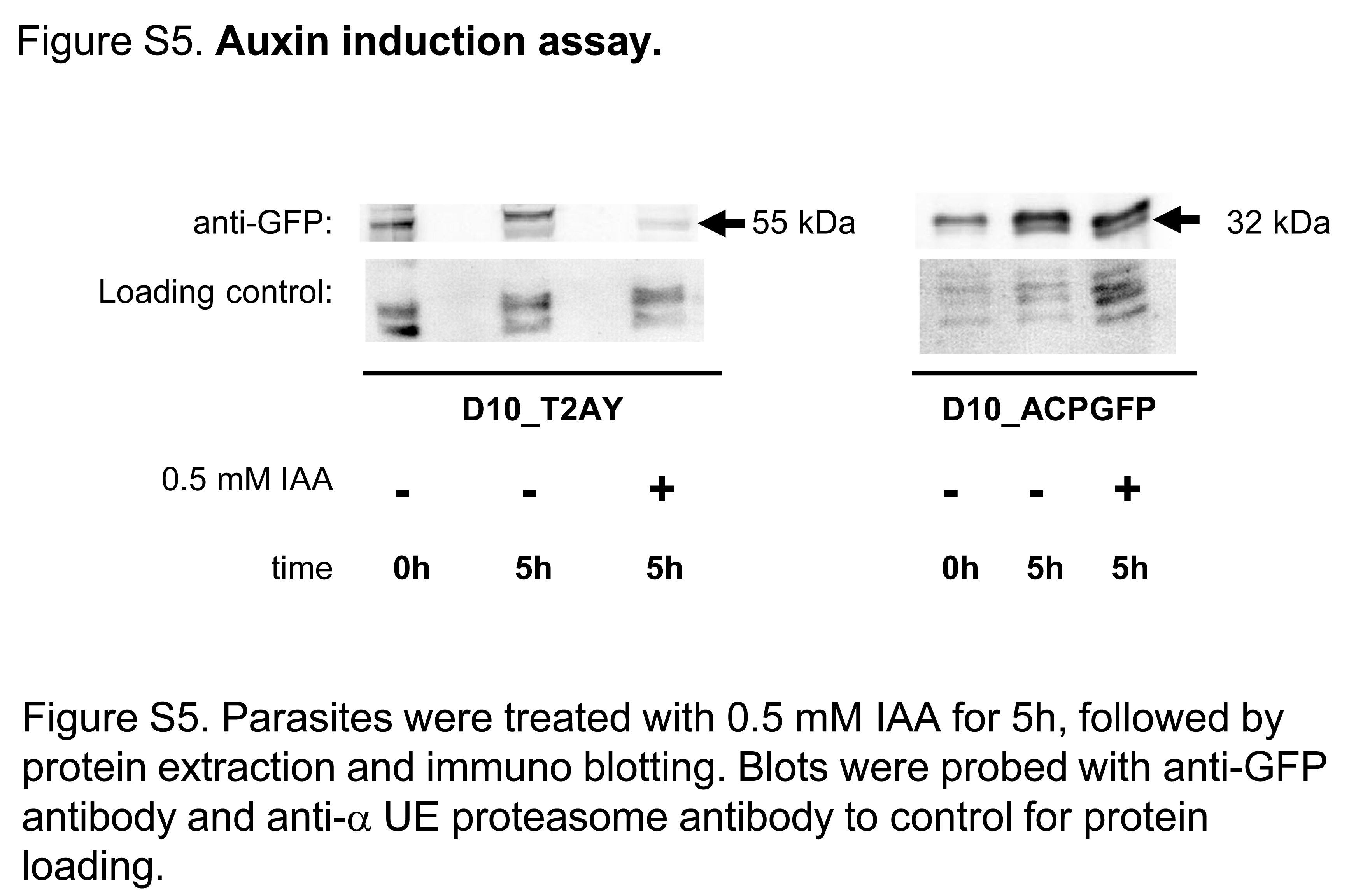

Supplement: Figure S5 — Auxin induction assay. (TIF) [file pone.0078661.s005.tif]

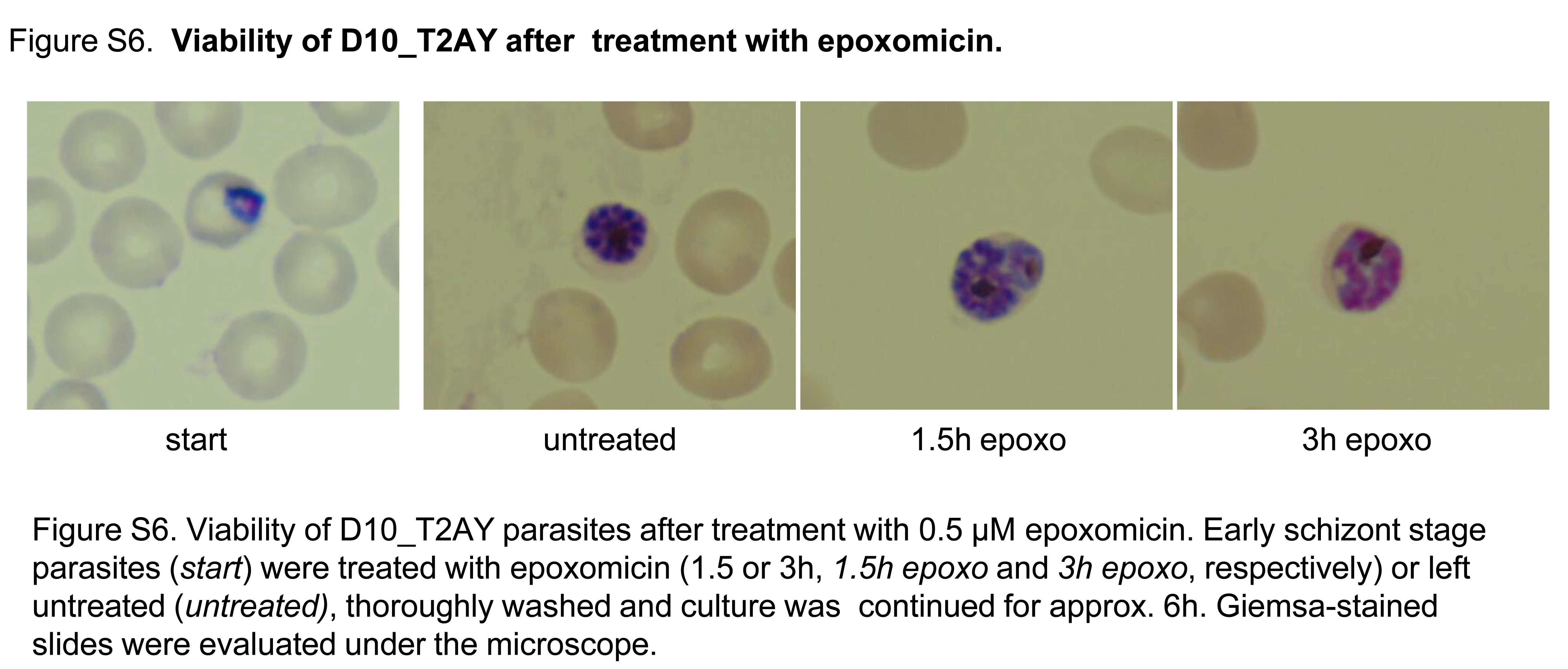

Supplement: Figure S6 — Viability of D10_T2AY after treatment with epoxomicin. (TIF) [file pone.0078661.s006.tif]
